# Association between psoriasis and oral pemphigus vulgaris: Report of two cases

**DOI:** 10.4317/jced.63181

**Published:** 2026-01-28

**Authors:** Débora Rosa Medeiros, Camila Silvério Carvalho Vieira, João César Guimarães Henriques, Mirna Scalon Cordeiro, Paulo Rogério de Faria, Sérgio Vitorino Cardoso, Carla Silva Siqueira

**Affiliations:** 1 Graduate Student, Dentistry School, Federal University of Uberlândia, Uberlândia, Minas Gerais, Brazil; 2 Department of Oral Diagnosis, Dentistry School, Federal University of Uberlândia, Uberlândia, Minas Gerais, Brazil; 3 Department of Oral Pathology, Dentistry School, Federal University of Uberlândia, Uberlândia, Minas Gerais, Brazil

## Abstract

The aim of this study is to illustrate the coexistence of these autoimmune conditions in the same patient, through the presentation of two case reports. Clinical Case 1: Male patient, fair-skinned, 43 years old, presenting with ulcerated and disseminated oral lesions for approximately six months, along with scaly lesions on the neck and elbows, previously diagnosed as psoriasis. An oral biopsy confirmed a diagnosis consistent with pemphigus vulgaris. Clinical Case 2: A 51-year-old fair-skinned male reported pain in the inner region of the mouth and throat for the past four months, associated with widespread ulcerative lesions. In addition, he presented scaly areas on the scalp. The findings confirmed a concomitant diagnosis of pemphigus vulgaris and psoriasis. In all cases, systemic corticosteroid therapy was prescribed, and the patients remain under joint follow-up care in the of Stomatology and Dermatology. Therefore, a thorough investigation through clinical, laboratory, and histopathological examinations is indispensable for the proper management of these conditions, ensuring effective treatment.

## Introduction

Psoriasis is an autoimmune skin disease caused by the hyperproliferation of keratinocytes in the epidermis, and it is estimated that 2% to 3% of the world population is affected by this disease, with equal incidence between the sexes (1 , 2). Although the pathogenesis of this inflammatory disorder remains unknown, the combination of genetic, environmental and immune factors is believed to be responsible for the disease arising (3). Clinically, psoriasis can be classified as vulgaris, erythrodermic, guttate, and pustular (4), and its manifestation depends on this division. Psoriasis has been reported to coexist with autoimmune blistering diseases (ABBDs), particularly bullous pemphigoid (BP) and pemphigus. These chronic autoimmune disorders primarily affect the skin and mucous membranes (4). While psoriasis and ABBDs are characterized by distinct pathophysiological mechanisms, evidence suggests a shared immunological basis that may their comorbidity and overlapping clinical features (4). The aim of this study is to provide a concise overview of the association between psoriasis and AIBDs through the presentation of two case reports that illustrate the coexistence of these conditions.

## Case Report

**Figure 1:**
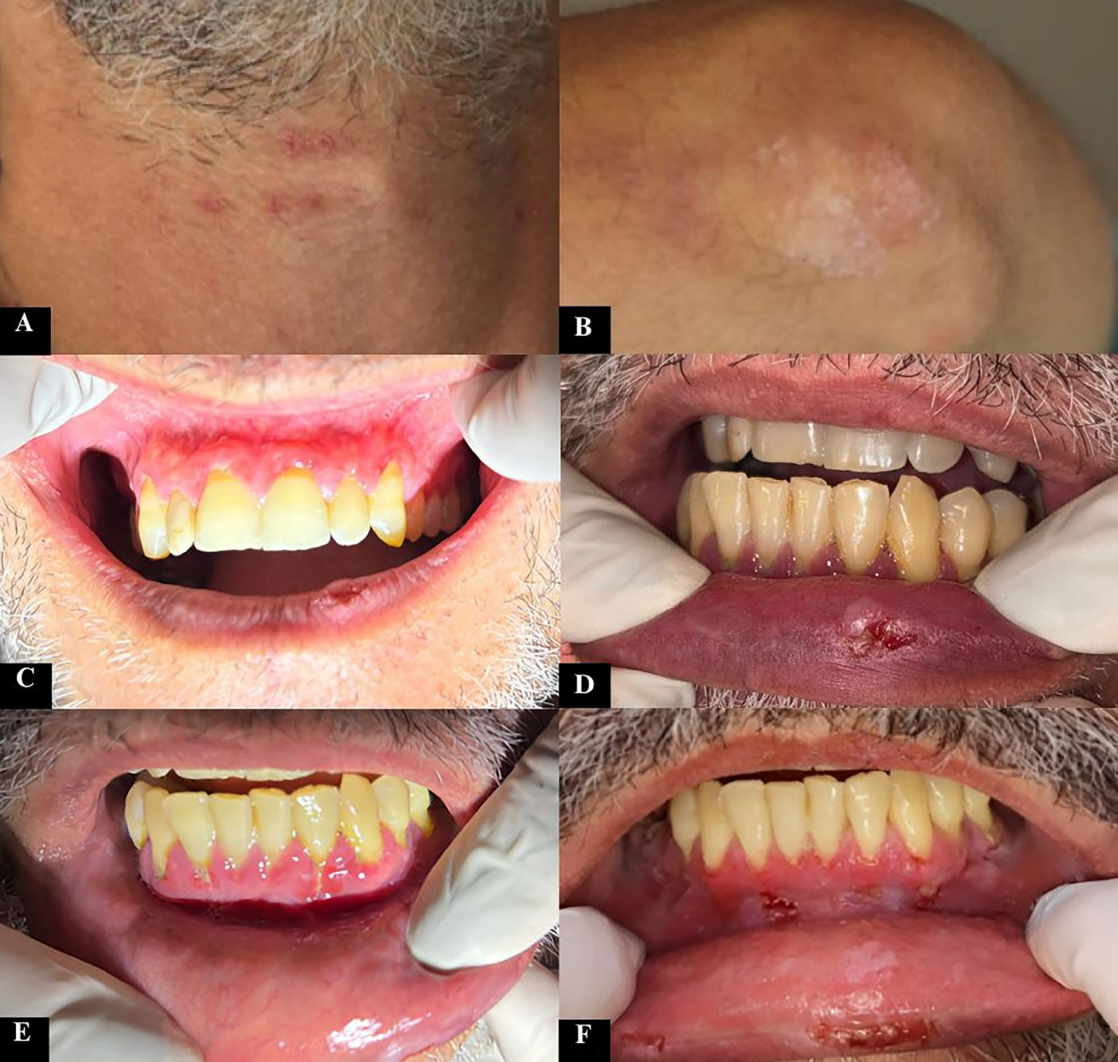
Images from extra- and intraoral assessments. A) Reddish lesions on the anterior cervical region. B) Reddish areas associated with silvery-white papules on the elbow. C) Erythematous superior alveolar mucosa. D) Ulcerated lesion on the lower lip. E) Desquamative gingivitis associated with the inferior anterior teeth. F) Improvement of the desquamative gingivitis after treatment with topical and systemic corticosteroids.

Case 1 A 43-year-old Caucasian male patient presented at the Stomatology Clinic of the Federal University of Uberlândia in 2021, complaining of widespread oral lesions that persisted for six months. During physical examination, it was noted that the patient presented red lesions on the anterior cervical region (Fig. 1A), along with silvery-white papules associated with reddish areas on both elbows (Fig. 1B).

[caption id="attachment_2043" align="alignnone" width="300"]1 Imagens artigo.pptx - 1[/caption]

**Figure 2:**
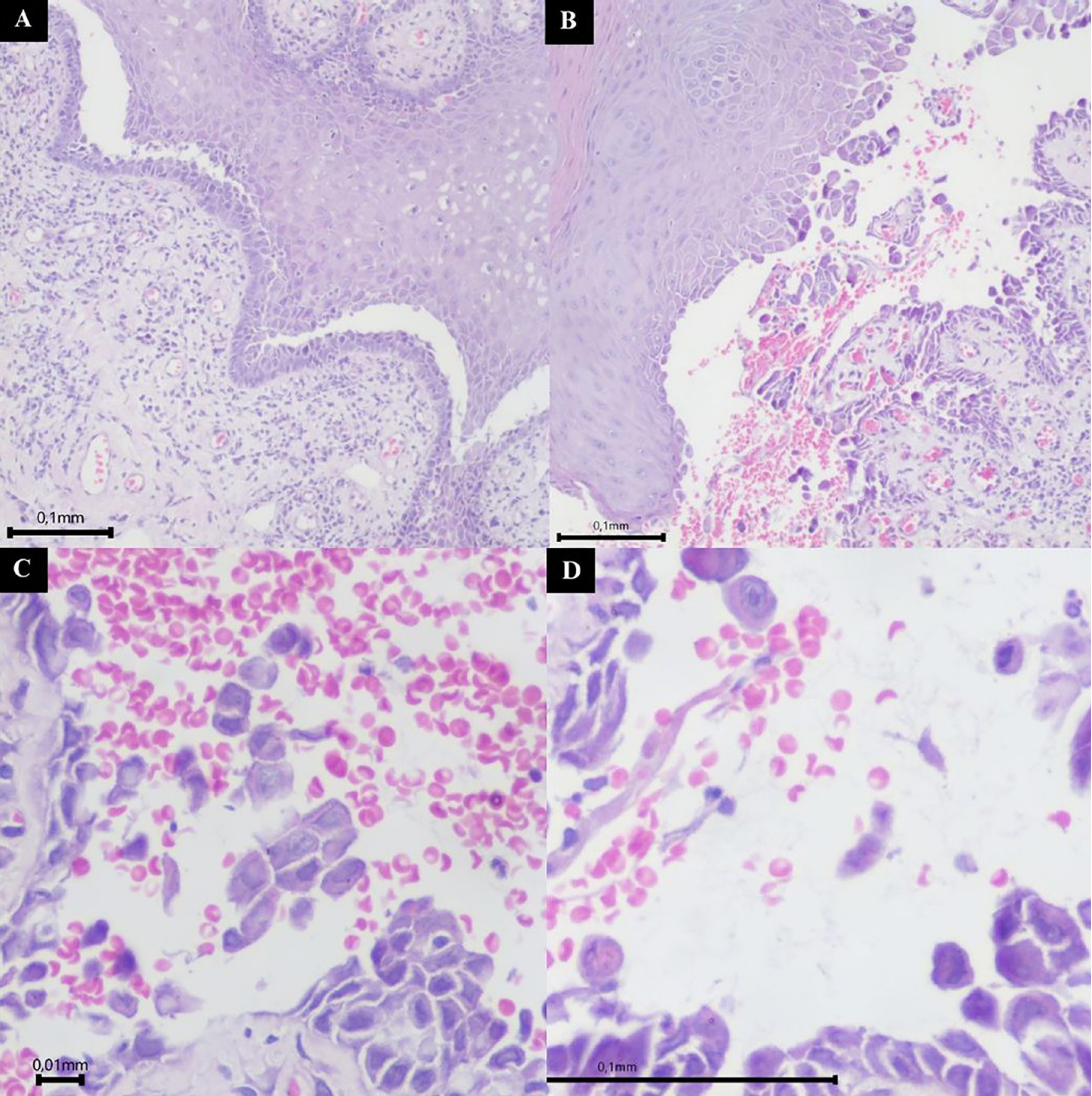
Microscopic view of the tissue fragment obtained from the incisional biopsy stained with hematoxylin and eosin. A) 10× magnification highlighting the separation of the epithelium from the connective tissue and a single remaining layer of epithelial cells. B) 10× magnification showing the intraepithelial blister. C) 40× magnification demonstrating acantholytic cells. D) 40× magnification demonstrating acantholytic cells.

The patient presented with clinical features consistent with cutaneous psoriasis, a condition previously diagnosed 27 years ago, as reported during the consultation. The patient has been under treatment and follow-up with a dermatologist, who made the initial diagnosis. On intraoral assessment, a widespread erythema was observed on the superior alveolar mucosa (Fig. 1C), an ulcerated lesion on the lower lip (Fig. 1D) and desquamative gingivitis associated with the inferior anterior teeth (Fig. 1E). An incisional biopsy revealed an intraepithelial blister with epithelial separation and a single row of cells adhered to the connective tissue (Fig. 2A,B), along with acantholytic cells (Fig. 2C,D), confirming the diagnosis of pemphigus vulgaris.

[caption id="attachment_2044" align="alignnone" width="300"]2 Imagens artigo.pptx - 2[/caption]

**Figure 3:**
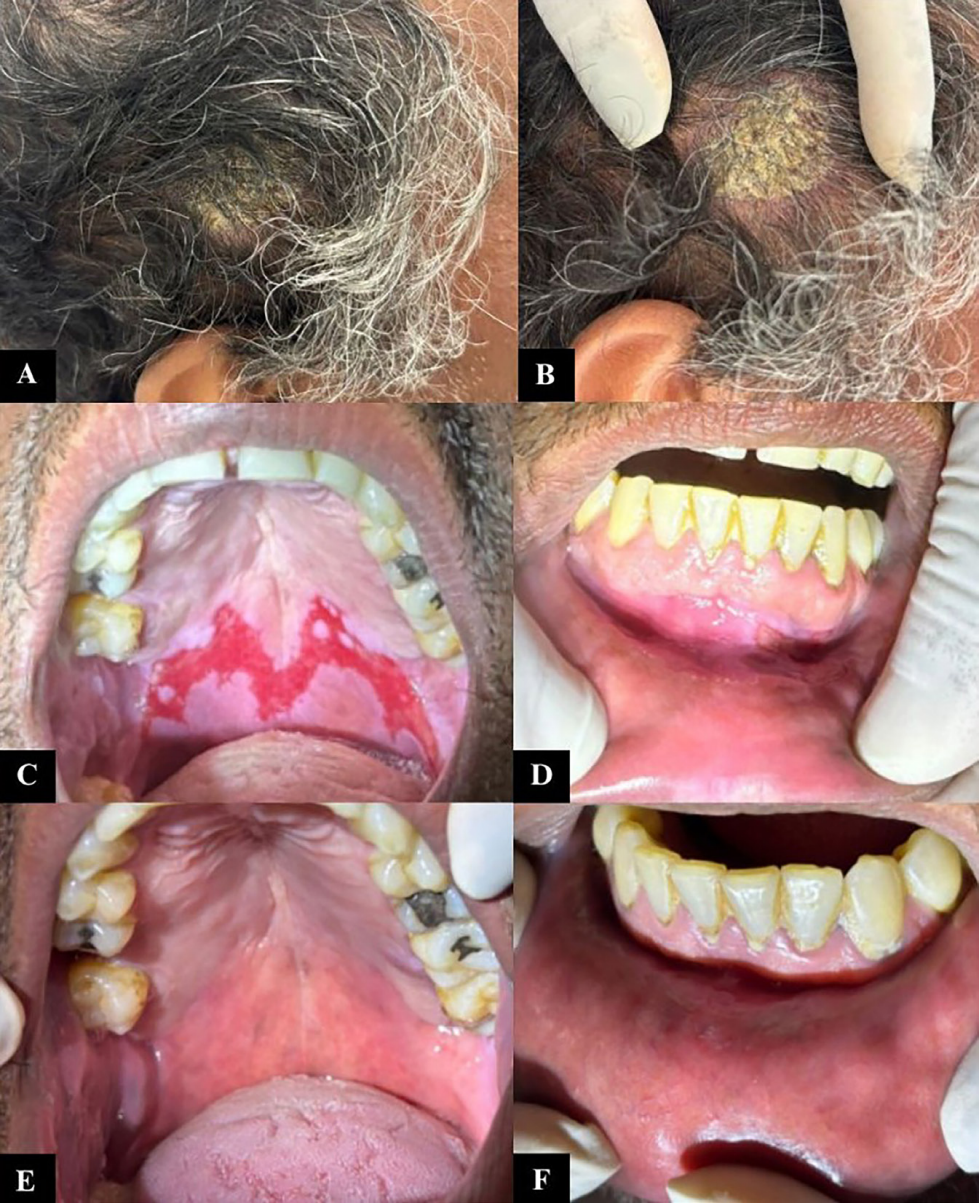
Images from extra- and intraoral assessments. A) Scaling area on the scalp. B) Scaling areas on the scalp. C) Ulcerated lesions on the soft and hard palate. D) Ulcerated lesion on the buccal mucosa. E) Improvement of the lesions on the palate after treatment with corticosteroids and immunosuppressant drugs. F) Improvement of the lesion on the buccal mucosa.

The patient was treated with oral corticosteroids (prednisone 40 mg) combined with topical betamethasone elixir 0.5 mg/5 mL, with subsequent gradual tapering of dosages, until significant improvement of the oral lesions, and received guidance on healthy habits. The patient was referred to the Dermatology Clinic at the same institution for follow-up of the dermatological lesions. He presented good clinical progression after treatment in both clinics. The patient underwent continuous dental outpatient monitoring for 2 years and 11 months. Case 2 A 51-year-old Caucasian male presented to the Stomatology Clinic at the Federal University of Uberlândia in 2024 reporting pain in the inner mouth and throat for the last four months. Clinical evaluation revealed ulcerated lesions on the soft and hard palate (Fig. 3A), as well as the left buccal mucosa (Fig. 3B), along with erythematous plaques with silvery-white scaling on the scalp, which the patient had not observed.

[caption id="attachment_2045" align="alignnone" width="244"]3 Imagens artigo.pptx - 5[/caption]

**Figure 4:**
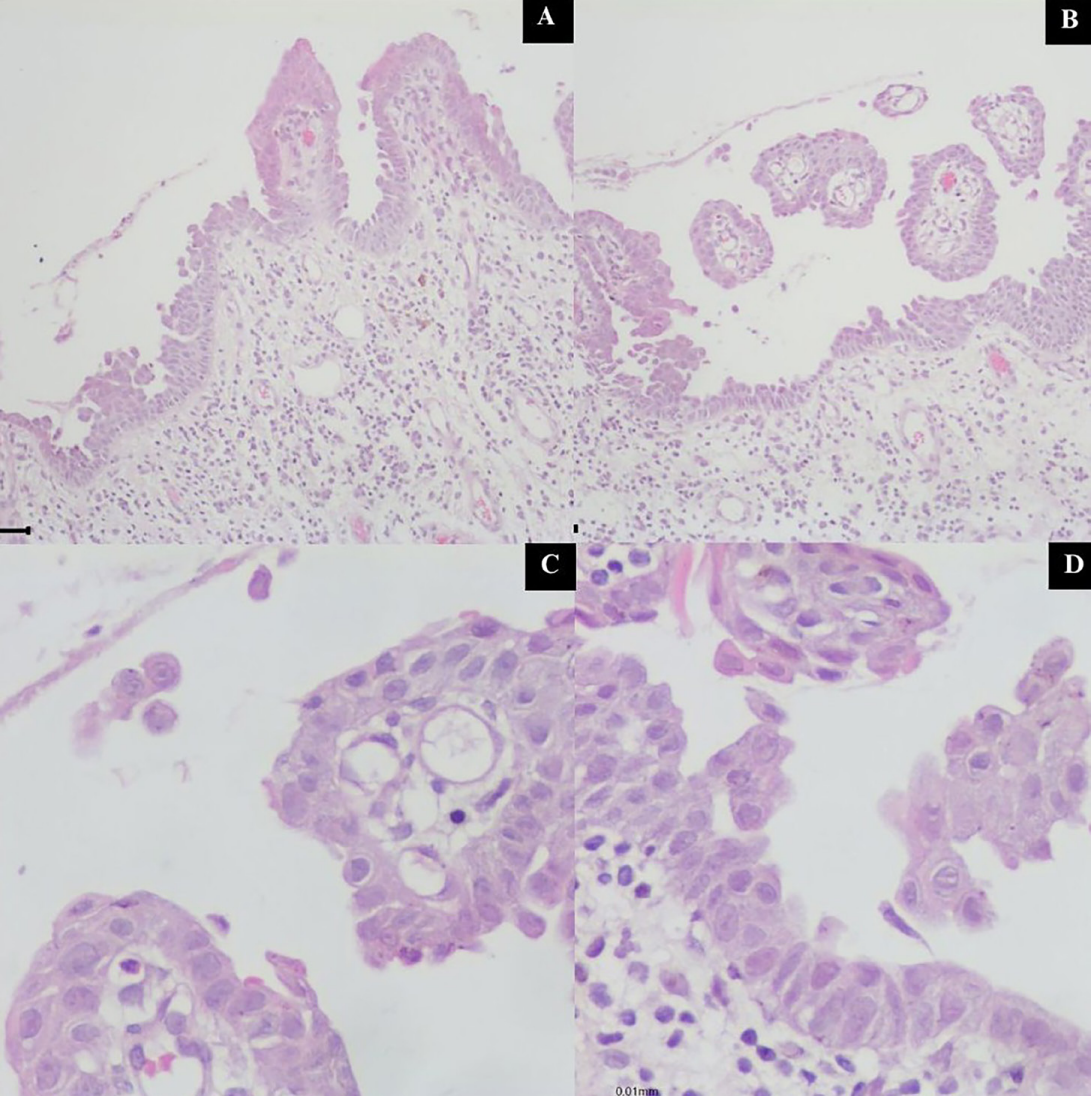
Microscopic view of the tissue fragment obtained from the incisional biopsy stained with hematoxylin and eosin. A) 10× magnification showing loss of the epithelium and a remaining layer of epithelial cells. B) 10× magnification showing loss of the epithelium and a remaining layer of epithelial cells. C) 40× magnification highlighting acantholytic cells. D) 40× magnification highlighting acantholytic cells.

A diagnostic hypothesis of autoimmune disease was raised, and incisional biopsy was carried out. On microscopic evaluation, loss of the epithelium was observed, and a single layer of epithelial cells was attached to the connective tissue, indicating the likely pre-existence of an intraepithelial blister (Fig. 4A,B). Acantholytic cells were also visualized (Fig. 4C,D).

[caption id="attachment_2046" align="alignnone" width="294"]4 Imagens artigo.pptx - 6[/caption]

In view of these findings, a diagnosis of Oral Pemphigus Vulgaris was set. Due to the dermatological features presented, the patient was referred to the Dermatology Service at the Federal University of Uberlândia for evaluation, where the diagnosis of psoriasis was confirmed. Since then, the patient has continued to receive care at the Stomatology Outpatient Clinic and the Dermatology Department, both at the Federal University of Uberlândia. In collaboration with the medical team, both oral and cutaneous lesions were treated with oral corticosteroids (prednisone 20mg) and azathioprine 50mg. Following lesion stabilization, gradual dose tapering was implemented, with the patient demonstrating favorable clinical improvement of the lesions (Fig. 3C,D). The patient remained under outpatient follow-up for one year.

## Discussion

Psoriasis is an autoimmune disorder with an estimated prevalence of 2% to 3% worldwide (1 , 2). Various studies have shown the association between this disease and bullous disorders such as Pemphigus Vulgaris (4 - 6). The present study aimed to report two cases of psoriasis associated with autoimmune diseases that presented oral manifestations. Regarding epidemiological features, both patients were Caucasian men over the fifth decade of life. Some studies have investigated the association between psoriasis and bullous disorders and have shown that these diseases significantly cooccur in men more often (5 , 6), even though pemphigus alone is more common in women (7) and psoriasis alone has no unanimous gender predilection (8). From the perspective of ethnic characteristics, psoriasis is known to be more frequent in white people (8) as shown in the present cases. The age of the patients from the present study is lower than reported by the literature, which reports the coexistence of psoriasis and pemphigus in individuals with mean ages ranging from 58 to 74 years (1 - 3). Clinically, the presentation of pemphigus was as reported by the literature, with erosion and ulceration especially in the cheek mucosa (6 , 7). The pathomechanisms underlying the psoriasis and pemphigus association have not yet been identified, but several hypotheses have been proposed, such the so-called "epitope spreading" phenomenon, with tissue injury secondary to a primary inflammatory process leading to the exposure of sequestered antigens evoking a secondary autoimmune disease (9). Corticosteroids, both topical and systemic, represent the first-line treatment for autoimmune diseases due to their proven efficacy in modulating the inflammatory and immune responses associated with these conditions (4). Due to the side effects of long-term drug use, alternative therapies, especially biologics targeting IL-23 and IL-17A like secukinumab and rituximab are being explored for treating psoriasis linked to pemphigus vulgaris, showing promising results. (10). However, further studies are needed to establish their long-term safety and effectiveness. Conventional immunomodulators, such as systemic methotrexate and topical tacrolimus, remain important therapeutic options, particularly in settings where access to targeted therapies is limited (4). Nevertheless, as each therapeutic decision must be individualized and the treatment should be approached in a multidisciplinary manner. In the present case report, all cases responded well to oral and topical corticosteroids, with a gradual reduction in dosage.

## Conclusions

The presence of oral mucosal lesions, often nonspecific and clinically challenging to diagnose, requires a multidisciplinary approach. Correlating clinical findings with laboratory and histopathological results is essential for establishing effective treatment strategies, leading to lesion improvement and enhanced patient quality of life.

## Data Availability

The datasets used and/or analyzed during the current study are available from the corresponding author.
